# Microwave-Assisted Synthesis of Glutathione-Capped CdTe/CdSe Near-Infrared Quantum Dots for Cell Imaging

**DOI:** 10.3390/ijms160511500

**Published:** 2015-05-19

**Authors:** Xiaogang Chen, Liang Li, Yongxian Lai, Jianna Yan, Yichen Tang, Xiuli Wang

**Affiliations:** Department of Dermatologic Surgery, Shanghai Skin Disease Hospital, Shanghai 200443, China; E-Mails: Anycall118@hotmail.com (X.C.); jackeylee198266@hotmail.com (L.L.); laiyongxian@gmail.com (Y.L.); everything5254@163.com (J.Y.)

**Keywords:** biomaterials, semiconductors, near-infrared, core/shell, quantum dots, glutathione, molecular imaging

## Abstract

These glutathione (GSH)-conjugated CdTe/CdSe core/shell quantum dot (QD) nanoparticles in aqueous solution were synthesized using a microwave-assisted approach. The prepared type II core/shell QD nanoparticles were characterized by UV–Vis absorption, photoluminescence (PL) spectroscopy, X-ray powder diffraction (XRD) and high-resolution transmission electron microscopy (HR-TEM). Results revealed that the QD nanoparticles exhibited good dispersity, a uniform size distribution and tunable fluorescence emission in the near-infrared (NIR) region. In addition, these nanoparticles exhibited good biocompatibility and photoluminescence in cell imaging. In particular, this type of core/shell NIR QDs may have potential applications in molecular imaging.

## 1. Introduction

Semiconductor quantum dots (QDs) have emerged as a new type of fluorescent material for bioimaging and have become very attractive in recent years due to their unique properties and advantages over traditional organic fluorophores [1]. The extreme brightness of QDs and their resistance to photobleaching make them ideal for live cell imaging [2]. In addition, the wide availability of precursors, controllable synthesis methods, and size-tunable emission ranging from ultraviolet [3] to near-infrared (NIR) regions [4,5,6] further expand the range of applications of QDs in molecular imaging.

To date, a number of NIR-emitting QDs, such as CdTe/CdS [7], CdHgTe [8,9,10], CdTeS [11], CdTeSe [12], and CulnS_2_ [13] QDs, have been successfully synthesized. Among them, type II core-shell QDs are often used because the physically separated electrons and holes in these structures easily result in emission in the NIR region, which corresponds to the range of optical transparency for living tissue [14]. However, these QDs suffer from limitations due to the toxicity of heavy metals in living cells and tissues. Surface coating has been widely used to prevent QD oxidation and thereby reduce the particles’ cytotoxicity. Some studies have reported the direct use of biomolecules as a capping agent for nanoparticles [15,16,17,18,19,20]. The advantage of this method is the simplicity of synthesis and firm linking of protein to the QDs surface. For example, glutathione (GSH) used for coating QDs can provide a physical barrier to the release of heavy metals, thus, GSH capped QDs showed little toxicity on living cells [21]. Recently, microwave irradiation was employed as a powerful heating system to enhance the quantum yield and water-soluble stability of these GSH capped QDs [22,23,24].

Herein, we report the facile synthesis of GSH-stabilized CdTe/CdSe QDs in aqueous solution using a microwave-assisted approach. The type II core-shell QDs exhibited emission in the NIR region with good biocompatibility and low cytotoxicity.

## 2. Results and Discussion

### 2.1. Characterization of Glutathione (GSH)-Stabilized CdTe/CdSe Quantum Dots (QDs)

**Figure 1:**
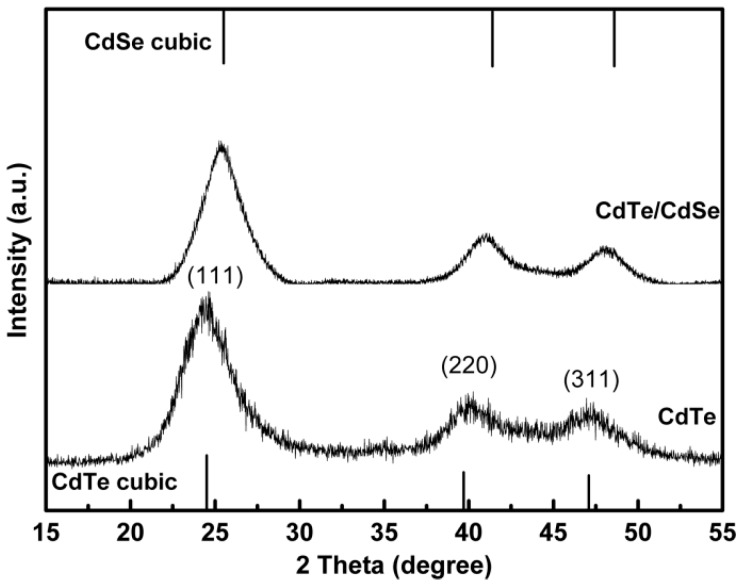
XRD diffraction patterns of CdTe and glutathione (GSH)-CdTe/CdSe quantum dots (QDs) prepared by microwave-assisted method.

Figure 1 shows the powder XRD pattern of the CdTe core and representative GSH-CdTe/CdSe core/shell QDs. The crystallinity for both two kinds of QDs was very high, and the broad diffractive peaks were due to their nanoscale sizes. The CdTe pattern was consistent with that of the cubic CdTe structure, the diffraction peaks corresponded to the (111), (220), and (311) crystal plane. When the CdSe shell was grown onto the CdTe core, the diffraction peaks of the XRD pattern moved to a higher angle while the peak widths and shapes were maintained, which clearly indicated the formation of GSH-CdTe/CdSe core/shell structure.

**Figure 2:**
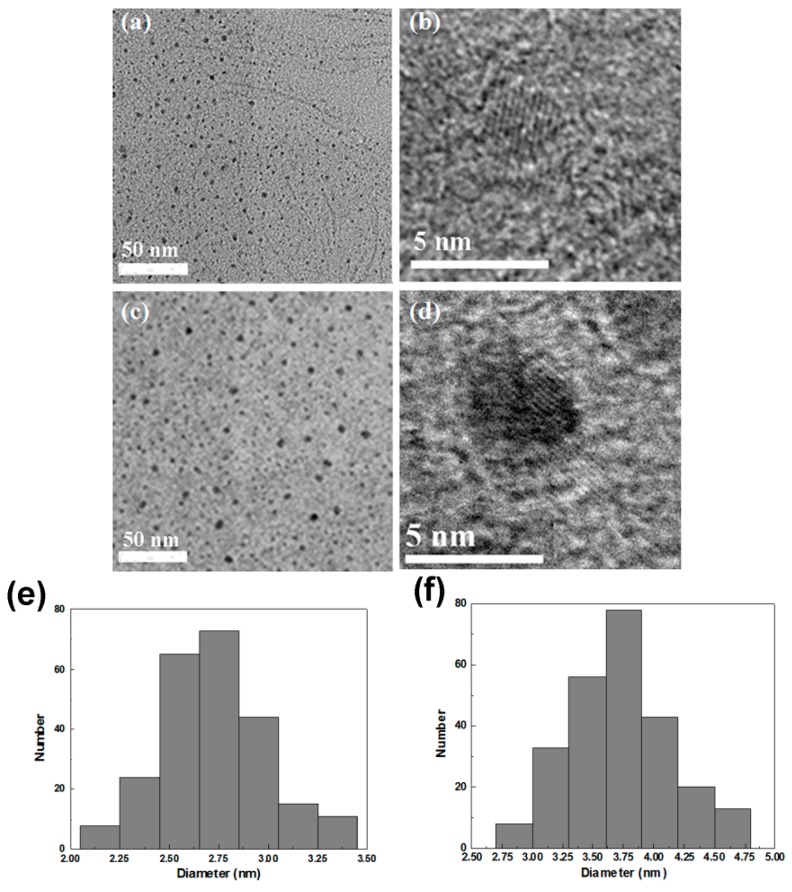
(**a**) Transmission electron microscopy (TEM) and high-resolution (HR)-TEM images of CdTe core (**a**,**b**) and GSH-CdTe/CdSe core/shell (**c**,**d**) QDs; Particle size distributions analysis of CdTe core (**e**) and GSH-CdTe/CdSe core/shell (**f**) QDs.

Figure 2 shows the TEM and HRTEM images of the CdTe core (a,b) and GSH-CdTe/CdSe core/shell (c,d) QDs. As seen in TEM images (Figure 2a,b), both the CdTe and GSH-CdTe/CdSe QDs had uniform sizes and good monodispersity. The well-resolved lattice fringe in HR-TEM images (Figure 2c,d) confirmed the good crystalline of both kinds of QDs. Moreover, as shown in Figure 2e,f, the average size and standard deviation of CdTe and CdTe/CdSe QDs were 2.75 ± 0.2 and 3.75 ± 0.3 nm, respectively. Additionally, the photoluminescence quantum yield (PLQY) of QDs at room temperature was estimated using previous reported method [25]. Thus, the PLQY of CdTe and CdTe/CdSe are 25% and 45%, respectively.

**Figure 3:**
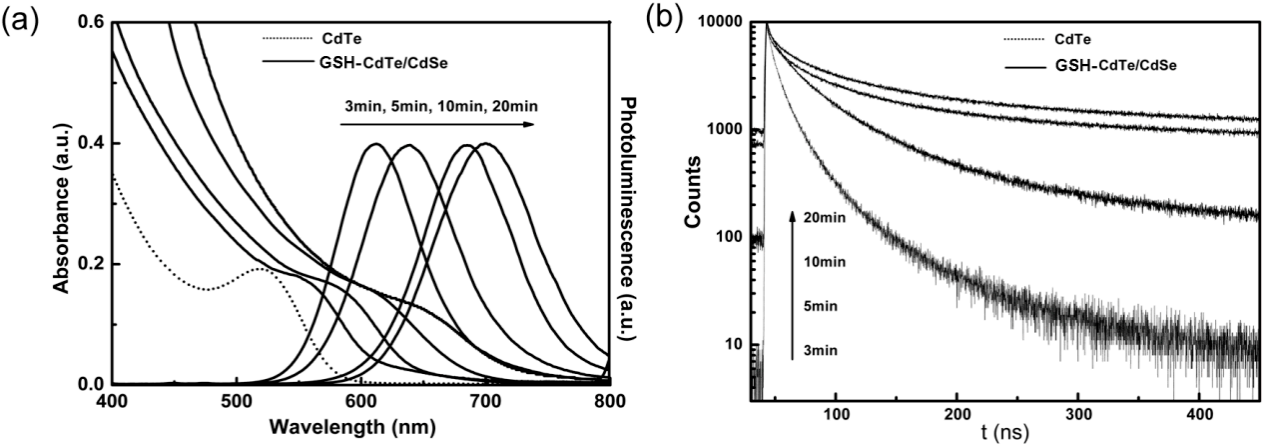
(**a**) UV–Vis and Photoluminescence spectra of CdTe (dashed line) and GSH-CdTe/CdSe QDs with different shell thickness (solid lines); (**b**) Fluorescence decay curves of a CdTe core (dashed line) and GSH-CdTe/CdSe QDs with different shell thickness (solid lines).

As shown in Figure 3a, in the UV–Vis absorption spectrum, the first excitoinc peak of the core/shell QDs shifted to longer wavelength and became gradually inconspicuous when the growth of the shell. This has been taken as a feature of type-II QDs with spatial separated charge carriers, which results from the weakened oscillator strength of the QDs due to the decreased wave function overlap [26,27]. In the PL spectrum, the CdTe QDs exhibited emission at 562 nm and no trap luminescence was detected. When the reaction time was up to 20 min, the emission wavelength shifted to 700 nm, with a significant increase of ~140 nm compared to the emission of the CdTe core QDs. A lifetime measurement is a typical method to determine the structure of type-II QDs. As seen in Figure 3b, according to Equation (1), the photoluminescence lifetimes of fresh prepared CdTe/CdSe QDs at 3, 5, 10 and 20 min is about 24.6, 32.3, 51.4 and 57.0 ns, respectively. Furthermore, a great increase of the decay lifetime was observed with the growth of the CdSe shell in Figure 3b. This is due to the spatial separation of electron and hole in type-II QDs [28,29] , which will result in a decrease of the wave function overlap and, thus, longer radioactive lifetime [30,31,32].

### 2.2. The Biocompatibility and Cell Imaging of GSH-CdTe/CdSe QDs

**Figure 4:**
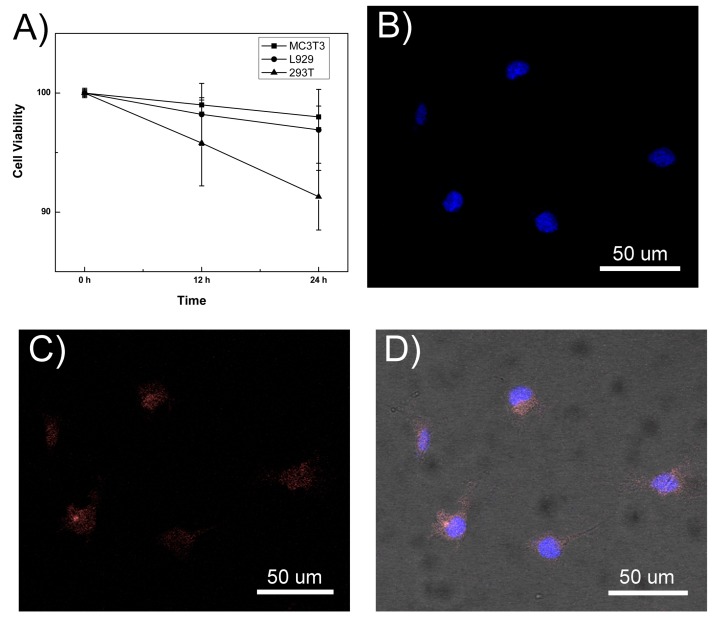
(**A**) Cell viabilities of mouse osteoblast precursor cell line MC3T3, mouse fibroblasts cell line L929 and human embryonic kidney cell line HEK293T incubated with RGD conjugated-GSH-CdTe/CdSe QDs at various time intervals from 12 to 24 h, respectively; (**B**–**D**) Representative laser scanning confocal microscope images of A375 cells incubated with RGD conjugated-GSH-CdTe/CdSe QDs for 3 h; The cells were stained by 4',6-diamidino-2-phenylindole (DAPI) (Blue, **B**), RGD conjugated-GSH-CdTe/CdSe QDs (Pink, **C**) and merge images (**D**); Original magnification of (**B**–**D**) is 200×.

The cytotoxicity effects of GSH-CdTe/CdSe QDs were further examined in three normal cell lines (MC-3T3, L929 and 293T) by using a standard 3-(4,5-cimethylthiazol-2-yl)-2,5-diphenyl tetrazolium bromide (MTT) assay. It was found that no evident cell proliferation inhibition was induced by GSH-CdTe/CdSe QDs in these three cell lines after 24- and 48-h treatment (Figure 4A). With increasing the incubation time to 48 h, GSH-CdTe/CdSe QDs only induced a limited decrease of cell viability (<10%) for 293T cells, indicating the good biocompatibility of GSH-CdTe/CdSe QDs against normal cells. For *in vitro* cell-labeling studies, these GSH-CdTe/CdSe QDs were conjugated with arginine-glycine-aspartic acid (RGD) cycle peptides, which is a common tumor biomarker for targeting tumor cells. As shown in Figure 4, the robust near-infrared (NIR) luminescent signals of RGD conjugated-GSH-CdTe/CdSe were detected from A375 cells. In addition, a control experiment was performed by incubating 293T cells with RGD conjugated-GSH-CdTe/CdSe QDs. Notably, there are no NIR luminescent signals observed in those cells (Date not shown), which confirmed the cell-targeting specificity of RGD conjugated-GSH-CdTe/CdSe QDs. In a word, the RGD peptide could enhance our prepared QDs targeting for tumor cells.

## 3. Experimental Section

### 3.1. Preparation of CdTe/CdSe Core/Shell Quantum Dots (QDs)

Firstly, monodispersed CdTe core QDs was prepared in aqueous solution by means of microwave-assisted synthesis. Typically, 3-mercaptopropionic acid was added to a N_2_-saturated 2.5 mM CdCl_2_ solution, followed by adjustment to the desired pH value (about 11) by the addition of 5 M NaOH solution. Then, a certain amount of freshly prepared NaHTe solution was added to the Cd-MPA (3-mercaptopropionic acid) solution. The molar ratio of Cd^2+^/MPA/HTe^−^ was set to 1:2.5:0.25. The precursor solution was subjected to microwave irradiation at 120 °C for about 2 min.

Secondly, CdTe/CdSe core/shell QDs were prepared in aqueous solution by means of microwave-assisted synthesis. Typically, GSH was added to an N_2_-saturated 2.5 mM CdCl_2_ solution, followed by adjustment to the desired pH value (about 11) by the addition of 5 M NaOH solution. Then freshly prepared NaHSe solution was added to the Cd-MPA solution, the molar ratio of Cd^2+^/MPA/HSe^−^ was 1:2.5:1. Then, 6 mL freshly prepared CdTe core QDs solution (not purified) was mixed with the N_2_-saturated CdSe precursor solution containg Cd^2+^, MPA and HSe^−^. The whole mixed solution was subjected to microwave irradiation at 60 °C for 3–20 min.

### 3.2. The Calculation of Photo Luminescence Quantum Yield (PLQY) and Photo Luminescence (PL) Lifetime

The PLQY of QDs at room temperature was estimated using standard method [33]. The optical density at the excitation wavelength of the Rhodamine 6G (R6G) and the QD samples in the solution were set to a similar value. The wavelength of the excitonic absorption peak of the QDs was set as the excitation wavelength for measurement. The integrated PL intensities of the QD and R6G were calculated from the fully corrected fluorescence spectrum. The PLQY of the QD samples was finally obtained by comparing the integrated PL intensities of the QDs and R6G. The PL lifetimes of QDs in this study were calculated by the following equation [30]:

(1) $$<\text{τ}>=\frac{{\text{α}}_{\text{1}}{{\text{τ}}_{1}}^{2}+{\text{α}}_{\text{2}}{{\text{τ}}_{2}}^{2}+{\text{α}}_{\text{3}}{{\text{τ}}_{3}}^{2}}{{\text{α}}_{\text{1}}{\text{τ}}_{1}+{\text{α}}_{\text{2}}{\text{τ}}_{2}+{\text{α}}_{\text{3}}{\text{τ}}_{3}}$$

### 3.3. Cell Viability and Proliferation Assays

Mouse osteoblast precursor cell line (MC3T3), Mouse fibroblasts cell line (L929) and human embryonic kidney cell line (HEK293T) were seeded at a density of 1 × 10^4^ cells per well in the 96-well plates, and then added with 50 μL of PBS and GSH-CdTe/CdSe QDs. After the additional 12- and 24-h incubation, cell survivals were measured using a tetrazolium salt MTT assay. Fresh growth medium (180 μL) and MTT (20 μL, 5 mg·mL^−1^) solution were added to each well. The plate was incubated for 4 h, and then 200 μL of dimethylsulfoxide (DMSO) was added to each well to dissolve the purple formazan crystals. Finally, the absorbance at 492 nm of each well was measure.

### 3.4. Preparation of RGD Conjugated GSH-CdTe/CdSe QDs and Cell Imaging

Ten microlitre of an aqueous solution of ethyl(dimethylaminopropyl) carbodiimide (EDC, 2 mg/mL) was added to 1 mL of the solution of GSH-coated CdTe/CdSe QDs prepared by the above method. Immediately after the addition, 20 μL of sulfo-NHS (*N*-hydroxysuccinimide) was added and vortexed. After 2 h, 100 μL of RGD cycle peptides (1 mg/mL) was added slowly, and the solution was incubated overnight at 4 °C. Human melanoma cancer A375 cells were plated at a density of 5 × 10^5^ cells per well in three 24-well plates with a glass cover slip bottom and incubated for 12 h at 37 °C with 5% CO_2_. RGD conjugated GSH-CdTe/CdSe QDs were subsequently added to the wells for incubation 3 h at 37 °C. Then, these cells were fixed by 5% paraformaldehyde and stained with 4',6-diamidino-2-phenylindole (DAPI). The cell imaging was analyzed by using an inverted confocal microscope (Leica TCS SP2 AOBS, California Nanosystems Institute, Heidelberg, Germany).

## 4. Conclusions

In summary, we developed a simple one-pot hydrothermal procedure for the synthesis of biocompatible NIR CdTe/CdSe QDs consisting of many GSH proteins assembled on their surface. The prepared GSH-CdTe/CdSe QDs demonstrated good water-solubility and dispensability, low cytotoxicity and strong NIR fluorescence, making them suitable for using as a NIR imaging agent for molecular imaging in future studies.
